# Myricetin Modulates Macrophage Polarization and Mitigates Liver Inflammation and Fibrosis in a Murine Model of Nonalcoholic Steatohepatitis

**DOI:** 10.3389/fmed.2020.00071

**Published:** 2020-03-04

**Authors:** Qunyan Yao, Shuyu Li, Xi Li, Fu Wang, Chuantao Tu

**Affiliations:** ^1^Department of Gastroenterology and Hepatology, Zhongshan Hospital, Fudan University, Shanghai, China; ^2^Shanghai Institute of Liver Diseases, Shanghai, China; ^3^Department of Geriatrics, Zhongshan Hospital, Fudan University, Shanghai, China; ^4^Shanghai Medical College, Fudan University, Shanghai, China; ^5^Department of Gastroenterology, Shanghai Public Health Clinical Center, Fudan University, Shanghai, China

**Keywords:** NASH, hepatic fibrosis, myricetin, macrophage polarization, TREM-1, TLRs, MyD88

## Abstract

This study aimed to investigate the beneficial effects of myricetin in a diet-induced nonalcoholic steatohepatitis (NASH) model and the underlying mechanism. C57BL/6J mice were fed a standard chow or the choline-deficient, L-amino acid-defined, high-fat diet (CDAHFD) for 8 weeks with the treatment of myricetin (100 mg/kg) or vehicle by daily gavage. Hepatic inflammation, steatosis, fibrosis, and hepatic stellate cells (HSC) activation were assessed. We also analyzed M1 and M2 macrophages and its related markers in livers from NASH mice and in RAW264.7 macrophages stimulated by lipopolysaccharide (LPS) or interleukin 4 (IL-4) *in vitro*. Furthermore, we determined the effect of myricetin on the triggering receptor expressed on myeloid cells-1 (TREM-1), toll like receptor (TLR) 2 and 4, and myeloid differentiation factor 88 (MyD88) signaling both in livers from mice and in RAW264.7 cells stimulated by LPS. Our results revealed that myricetin remarkably ameliorated hepatic steatosis, inflammation, and inhibited hepatic macrophage infiltration in CDAHFD-fed mice. Myricetin-treated to CDAHFD-fed mice also inhibited liver fibrosis and HSC activation when compared with vehicle-treated to those mice. Moreover, myricetin inhibited M1 macrophage polarization and its relative markers in livers of NASH mice while induced M2 polarization. Similarly, *in vitro* study, myricetin inhibited the LPS-induced mRNA expression of M1 macrophages marker genes and induced IL-4-induced M2 macrophage marker genes in RAW264.7 macrophages. Mechanically, myricetin inhibited the expression of TREM-1 and TLR2/4-MyD88 signaling molecules in livers from NASH mice and in RAW264.7 macrophages stimulated by LPS *in vitro*. Additionally, myricetin inhibited the activation of nuclear factor (NF)-κB signaling and the phosphorylation of the signal transducer and activation of transcription 3 (STAT3) in LPS-stimulated RAW264.7 macrophages. Taken together, our data indicated that myricetin modulated the polarization of macrophages via inhibiting the TREM-1-TLR2/4-MyD88 signaling molecules in macrophages and therefore mitigated NASH and hepatic fibrosis in the CDAHFD-diet-induced NASH model in mice.

## Introduction

Nonalcoholic fatty liver disease (NAFLD) has recently emerged as a significantly public health issue because of its high prevalence (1–3). NAFLD is characterized by a wide spectrum of liver phenotypes ranging from simple steatosis to nonalcoholic steatohepatitis (NASH) (1–3). The most important clinical challenge in NASH is the progression to liver fibrogenesis, which may gradually develop to cirrhosis and eventually to hepatocellular carcinoma (HCC) (1–3). However, the molecular mechanisms underlying NAFLD onset and progression remain poorly understood, and there is currently no approved pharmacological therapy for NASH and fibrosis (1, 2). Therefore, a better understanding of the mechanisms of NASH development and progression is indispensable for identifying novel therapeutic strategies for this burgeoning hepatic disease.

Recently, it has become apparent that liver-resident macrophages and recruited macrophages play an important role in the development and progression of NASH and liver fibrosis (4–7). Macrophages are highly plastic cells that can shift to adapt to tissue microenvironment, which have different functional phenotypes with proinflammatory M1 macrophages and anti-inflammatory M2 macrophages (4, 7, 8). Moreover, M1-polarized macrophages exacerbate hepatic injury and inflammation through production of proinflammatory cytokines, such as tumor necrosis factor (TNF)-α and interleukin (IL)-1β, while M2 polarity switch of macrophages could inhibit the activation of M1 macrophages through secreting anti-inflammatory cytokines, including IL-10 (4–9). Notably, there is growing evidence that M1 macrophages can promote disease development and progression in NASH (4, 7). In contrast, pharmacological alteration of polarization from M1 to M2 phenotype partially has ameliorated the pathogenesis of steatohepatitis and fibrosis (7–9). Those data suggest that the switch in macrophage phenotypes determines their role in liver inflammation and fibrosis, and thus regulating the polarization of macrophage by modulating the key macrophage transcription factors represents therapeutic targets for NASH and liver fibrosis (8–12).

The triggering receptor expressed on myeloid cells (TREM)-1 is a kind of immunoglobulin superfamily activation receptors express on neutrophils and monocyte macrophages (13, 14). Upon activation, TREM-1 can trigger and augment inflammatory reaction, especially through synergism with toll-like receptors (TLRs) signaling in macrophages (13–16). Moreover, several studies have already revealed that TREM-1 play an important role in regulating the activation of Kupffer cell and is associated with macrophages polarization, which amplifies acute and chronic inflammatory responses in diseases (17–19). Interestingly, a recent report has demonstrated that overexpression of TREM-1 in the liver and M1 hepatic macrophages polarization were associated with obesity-induced insulin resistance (IR) (20). Additionally, in patients with metabolic syndrome, there was elevated levels of free fatty acids and lipopolysaccharide (LPS), which can stimulate TREM-1 expression and activate TLRs receptor cascade in lipid rafts (21). On the other hands, it is well known that TLRs-mediated signals are implicated in the pathogenesis of chronic liver diseases (22–24). Importantly, previous studies have revealed that TLR2/4-mediated myeloid differentiation factor 88 (MyD88) and nuclear factor-κB (NF-κB) signaling regulated macrophages polarization (20, 25, 26). Thus, we speculate that the TREM-1-TLR2/4-MyD88 signaling pathway may promote hepatic inflammation and fibrosis in NASH via modulation of macrophage polarization.

**Figure 1:**
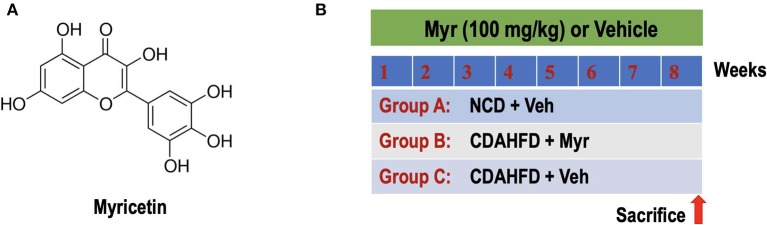
Experimental study design. **(A)** The chemical structure of myricetin (3,3',4',5,5',7-hexahydroxyflavone). **(B)** Experimental protocol for assessment the preventive effect of myricetin (Myr) on the development of NASH and fibrosis in mice fed the choline-deficient, L-amino acid-defined, high-fat diet (CDAHFD). Group A: control mice fed normal chow diet (NCD) and treated with the vehicle (Veh, 0.5% CMC-Na). Group B or C: CDAHFD-induced-NASH mice were randomly assigned to a treatment of Myr (100 mg/kg) or Veh by daily orally administration for 8 weeks.

Myricetin (3,3′,4′,5,5′,7-hexahydroxyflavone; Figure 1A) is a polyphenol flavonoid that is widely found in most berries such as blueberries and strawberries, tea, vegetables, and various medicinal herbs (27–29). Myricetin is reported to possess many pharmacological properties, including antioxidant, anti-inflammatory, antifibrotic, anti-obesity, and anti-diabetic activities (27–34). Recent studies have also demonstrated that myricetin attenuated vascular endothelial dysfunction and hepatic injury in mice induced by high choline-fed (31) and mitigated liver fibrosis induced by CCl_4_ in mice (29, 32). In particular, myricetin has proved to inhibit fatty acid biosynthesis and attenuate ethanol-induced lipid accumulation in liver cells (30). Furthermore, myricetin treatment also ameliorates hyperglycemia, IR, and steatosis in mouse models of obesity through antioxidant properties (33, 34). These findings indicate that supplementation of the diet with myricetin might be beneficial to NASH and liver fibrosis. Therefore, the purpose of this study is to investigate whether myricetin could attenuate NASH-related inflammation and fibrosis in a mouse model of NASH and to elucidate its underlying mechanisms; we particularly focus and assess the effects of myricetin on macrophages polarization.

## Materials and Methods

### Reagents and Antibodies

Myricetin was purchased from Selleck Chemicals Co. Ltd. (Houston, TX, USA). Sodium carboxymethyl cellulose (CMC-Na) was obtained from Makclin Biochemical Co., Ltd. (Shanghai, China). Dimethyl sulfoxide (DMSO) and lipopolysaccharide (LPS) were purchased from Sigma Chemical, Co. Ltd. (St. Louis, MO, USA). Myricetin was prepared as stocks in CMC-Na and diluted to various concentrations in medium.

The antibodies were obtained as following: rabbit anti-IL-12A, rabbit anti-IRF5, rabbit anti-CD163, rabbit anti-Ym-1/Ym-2, rat anti-TREM1, rabbit anti-NF-κB, rabbit anti-phosphor-NF-κB (Abcam, Cambridge, MA); rabbit anti-α-SMA, rabbit anti-F4/80, rabbit anti-Iκ-Bα, rabbit anti-phospho-Iκ-Bα, rabbit anti-JNK, rabbit anti-phospho-JNK, mouse anti-STAT3, rabbit anti-phospho-STAT3 (CST, Danvers, Massachusetts, USA); HRP-linked goat anti-rabbit IgG (Jackson Labs Technologies, Inc., Las Vegas, NV); rabbit anti-GAPDH and rabbit anti-β-actin (HuaAn Technologies, Inc., Hangzhou, China). HRP-linked goat anti-rat IgG (Byotime Institute Biotechnology, Shanghai, China), Cy3 conjugated goat anti-rabbit IgG H&L (Servicebio, Wuhan, China), Alexa Fluor 488 goat anti-rat IgG H&L (Invitrogen, USA).

### Animals and Experimental Protocols

Six-week-old male C57BL/6J mice (20–23 g) were purchased from Shanghai Slack laboratory animal co., Ltd. (Shanghai, China). Mice were maintained in a temperature-controlled room (23 ± 3, 55 ± 10% humidity) with a 12-h light-dark cycle and had unrestricted to normal chow diet (NCD) or a choline-deficient, L-amino acid-defined, high-fat diet (CDAHFD) consisting of 60% kcal fat and 0.1% methionine (Research Diets, Inc., New Brunswick, NJ, USA) (35, 36). The mice were randomly divided into three groups (*N* = 8 for each group). Group A was given NCD and treated with vehicle (0.5% CMC-Na). Group B or C: mice were fed CDAHFD with orally administration myricetin at 100 mg/kg per day or vehicle (Figure 1B). The doses of myricetin were chosen based on previous studies in mice (28, 32, 37). At the end of treatment period, mice were euthanized using ketamine/xylazine, blood samples were collected via cardiac puncture to detect biochemical biomarkers. Livers were removed for measurement weight, photographed, and processed for further histological and molecular assessment. All samples were stored at −80°C until use. All animal experiments were performed according to the guidelines of the care and use of laboratory animals of Fudan University and approved by the Animal Ethics Committee of Zhongshan hospital.

### Culture and Treatment of RAW264.7 Macrophage Cells

RAW264.7 murine cells were purchased from the Cell Resource Center, Shanghai Institutes for Biological Sciences of the Chinese Academy of Sciences (Shanghai, China) and cultured in undifferentiated RAW macrophages conditioned medium as previously described (38, 39). Briefly, the cells cultured in T25 flasks in Dulbecco's modified Eagle's medium (DMEM) supplemented with 10% fetal bovine serum (FBS), glutamine (2 mM L), penicillin (50 U/mL), and streptomycin (50 μg/mL) at 37 and 5% CO_2_.

*In vitro* experiments evaluating the effect of myricetin on the activation and polarization of macrophages, RAW264.7 cells were polarized by culturing 10^6^ cells/well overnight in 6-well plates before replacing the conditioned-medium to induce M1- or M2-polarized macrophages as descripted previously (9, 11, 39). Briefly, cells were classically activated with 100 ng/mL LPS (M1 condition) or alternatively activated with M2 condition (20 ng/mL IL-4), respectively; control cells were cultured with DMEM alone (M0 condition). For selective experiments, cells were pretreated with myricetin (50 μM) or vehicle (0.5% DMSO) for 12 h, then cells were added the macrophages conditioned medium for another 12 h. Finally, cells were then washed and harvested by centrifugation for immunofluorescence analysis, RNA and protein analysis. All measurements were performed in triplicate wells. For cells experiment, a stock myricetin solution (10 mM) was prepared using DMSO as the solvent and stored at −20 until use. Myricetin concentration for cells treatment was based on our primary study and previous *in vitro* bioactivity work (29, 40, 41).

### Cell Viability Assays

RAW264.7 cells viability was evaluated by the Cell Counting Kit-8 (CCK8)-based spectrophotometric methods (Beyotime Institute Biotechnology, Shanghai, China) according to the protocol provided by the manufacturer. Cells were seeded in 96-well flat-bottom plates at a density of 5 × 10^3^ cells/well. After 6 h of culture, the medium was then changed to serum-free medium containing 0.5% DMSO (vehicle) or various concentrations of myricetin (0, 25, 50, and 100 μM) for 0, 12, 24, or 48 h at 37 and 5% CO_2_. Following treatment, 10ul CCK8 solution was added in each cell and incubated for another 2 h at 37. Relative cytotoxicity was measured at 450 nm absorbance with Biotek EPOCH2 microplate reader (BioTek Instruments Inc., USA). Cell viability was defined relative to the vehicle-treated control, and each experiment was done three times independently to ensure reproducible results.

### Serum Enzymes Assays

The serum alanine transaminase (ALT) and aspartate transaminase (AST) activity were analyzed using the kits from Nanjing Jiancheng Bioengineering Institute (Nanjing, China) respectively following the manufacture's standard protocol.

### Histopathology

Liver samples were collected from each mouse and fixed in 10% neutral buffered formalin and embedded in paraffin. Then these liver tissues were cut in 4-μm-thick sections and stained with hematoxylin and eosin (H&E), or Masson's trichrome according to standard procedures. Hepatic histopathological examination was performed in a blinded manner by an experienced pathologist with the histological scoring system for NAFLD (35, 42). Briefly, hepatocellular steatosis and liver inflammation scores were classified into grades 0 to 3 with 0 being within normal limits and 3 being most severe; the staging of liver fibrosis was classified into stages 0 to 4. Individual scores were assigned for each parameter. Moreover, liver fibrosis was also evaluating using the NIH ImageJ free software (Bethesda, Maryland, USA) on Masson's trichrome-stained sections in a blinded manner (23, 38).

### Oil Red O Staining

Lipid accumulation in the liver was evaluated using an Oil Red O (ORO) staining kit (Sigma Chemical, Co. Ltd., St. Louis, MO, USA) as described in the manufacturer's procedure. All images were obtained using an Observer A1 microscope (Carl Zeiss) at ×100 magnification. For quantification ORO-positive staining, 5 randomly non-overlapping ×100 fields per specimen were examined and determined for six animals in each group using the NIH ImageJ free software (Bethesda, Maryland, USA). Results are expressed as percentages of positive areas in the high-power field.

### Immunohistochemistry Staining and Analysis of Histological Markers

Immunohistochemistry (IHC) staining was carried out as our previously described (23). Briefly, formalin-fixed tissues were embedded in paraffin, cut 4-μm-thick sections. Followed by dewaxing, hydration and antigen retrieval by heat, sections were then blocked and incubated overnight at 4 with primary antibodies as follows: anti-SMA (1:100), anti-F4/80 (1:100), anti-CD163 (1:100), anti-Ym-1/Ym-2 (1:100), anti-IL-12 (1:100), and anti-IRF5 (1:100), with each primary antibody diluted in TBS containing 2% bovine serum albumin (BSA). Sections then were subsequently washed 3 times and incubated with HRP-conjugated goat anti-rabbit IgG secondary antibody, followed by incubation for 5- to 10-min with 3, 3′-diaminobenzidine tetrachloride and visualization of specific staining by light microscopy. Images were acquired under high-power field with Nikon Eclipse Ti inverted microscope (Nikon, Amstelveen, The Netherlands).

Quantitative expression of immunostaining was carried out at a fixed threshold using NIH ImageJ software (Bethesda, Maryland, USA). For quantification α-SMA-positive areas in liver section, five random non-overlapping ×100 fields were examined and determined for six animals in each group (38, 43). For quantification the areas of hepatic macrophages (F4/80^+^, IL-12^+^, IRF5^+^, Ym-1/Ym-2^+^, and CD163^+^ cells) in sections, six random non-overlapping selected fields of view per slide at ×200 magnifications were analyzed and expressed as the percentage of positive area in the high-power field; and five mice of each group were examined (44).

### TUNEL Staining

Terminal deoxynucleotidyl transferase mediated dUTP nick-end labeling (TUNEL) staining was carried out to evaluate death hepatocytes in the liver. The paraffin-embedded liver tissue sections were stained using a DeadEnd™ Fluorometric TUNEL System based on the manufacturer's protocol (Promega, USA). Sections were counterstained with 4′,6-diamidino-2-phenylindole (DAPI) and hematoxylin solution and observed with Nikon Eclipse Ti inverted microscope (Nikon, Amstelveen, The Netherlands). The TUNEL-positive nuclei (blue) were quantified under ×200 magnification in 5 randomly non-overlapping fields and 5 animals of each group were assessed (16). The results were presented as the mean number of TUNEL^+^ cells each field.

### Immunofluorescence and Quantification

The dissected liver tissues from mice were fixed in 4% paraformaldehyde, washed with PBS (pH 7.4), embedded in optimum cutting temperature tissue compound (OCT compound, Sakura, Japan), and stored at −80 for 24 h (16). Then the sections (8μm in thickness) were prepared with a cryotome Cryostat (Leica, CM 1900, Germany). After antigen retrieval was performed, blocking was carried out in PBS with 3 % BSA. Slides were incubated with primary antibody against F4/80 (dilution, 1:50) at 4°C overnight, then incubated with TREM-1 antibody (dilution 1:50) at RT for 1 h in case of double-staining. Cy3 conjugated goat anti-rabbit IgG H&L and Alexa Fluor 488 goat anti-rat IgG H&L antibodies were incubated at 1:200 in PBS at RT for 1 h. After washing with TBS for 3 times, the cell nuclei were counterstained with DAPI-Fluoromount-G^TM^ (Southern Biotech, USA). Finally, the stained tissues were analyzed by fluorescence microscopy (BX51, Olympus, Japan).

RAW264.7 cells were fixed with 4% paraformaldehyde for 15 min at RT followed by permeabilization using 0.2% TritonX-100 in PBS. Then nonspecific binding was blocked with 3% BSA for 1h at RT, followed by incubation with primary antibodies for IL12 (dilution 1/100) and IRF5 (dilution 1/100) at 4 overnight. After twice washing in PBS, cells were incubated with secondary antibody for 1 h at RT. DAPI was used for nuclear staining. The slides were washed twice with PBS, covered with DABCO (Sigma-Aldrich, St. Louis, MO), and imaged by fluorescence microscopy (IX51, Olympus, Japan). Percentage of the IL-12^+^ and IRF5^+^ staining area in 6 randomly selected fields using the NIH ImageJ free software (Bethesda, Maryland, USA).

### Western Blotting

Western blotting was performed as our described in detain previously (23, 43). In brief, homogenized liver tissue or harvested cells were lysed with RIPA buffer containing protease and phosphatase inhibitor cocktail (Thermo Scientific). The protein concentration in each was determined via the Bradford method (Bio Rad, Sydney, Australia). Forty microgram protein was separated by electrophoresis on a proper concentration of SDS-PAGE gels and transferred to nitrocellulose membranes. After blocking with 5% bovine serum albumin (BSA) in tris-buffered saline plus tween for 1 h and incubation with primary and secondary antibodies, the blots were visualized by ECL™ Western Blotting Detection Reagents (Amersham Pharmacia Biotech Inc., NJ, USA). The optical density of each band was quantified by NIH ImageJ free software (Bethesda, Maryland, USA) and normalized to GAPDH or β-actin as a loading control. The specific primary antibodies in our study were diluted as follows: α-SMA (1:1000), TREM-1 (1:500), IL-12A (1:1000), IRF-5 (1:1000), NF-κB (1:1000), phospho-NF-κB (1:1000), Iκ-Bα (1:1000), phospho-Iκ-Bα (1:1000), STAT3 (1:1000), phospho-STAT3(1:1000), JNK (1:1000), phosphor-JNK (1:1000), anti-GAPDH (1:1000), and anti-β-actin (1:1000).

### RNA Isolation and Quantitative RT-PCR Analysis

Total RNA was isolated from frozen snap-frozen mouse livers or RAW264.7 cells using the Trizol reagent (Life Technologies, Grand Island, NY) according to the manufacturer's protocol. Single-stranded cDNA was synthesized using random hexamer primers and avian myeloblastosis virus reverse transcriptase commercial kit (Perfect Real Time, SYBR® PrimeScriP™TaKaRa, Japan). Quantitative RT-PCR reactions were carried out for assessment of mRNA expression on an ABI Prism 7500 sequence detection system (Applied Biosystems, Tokyo, Japan) as descripted previously (23, 43). Relative gene expression was normalized to β-actin as housekeeping gene, and fold change over the untreated control was calculated using the 2^−ΔΔ^Ct method (23). The primers sequences of the target genes were purchased from Sangon Biotech Co., Ltd. (Shanghai, China) and were provided in Table S1.

### Statistical Analysis

All data are presented as mean ± standard error of the mean (SEM), unless otherwise stated. All statistical analyses were carried out using GraphPad Prism 8.0 software (La Jolla, CA, USA). Differences between multiple groups were compared by one-way analysis of variance (ANOVA) with *post hoc* test (Tukey's correction for multiple tests). For comparison between two groups, the unpaired two-tailed Student's *t*-tests were used. In case of non-normality in distribution Wilcoxon-Mann-Whitney *U*-tests or Kruskal-Wallis tests were used to compare quantitative data, as appropriate. For all tests, *P* < 0.05 were considered statistical significance and the level of significance was shown by asterisks (^****^P < 0.0001; ^***^P < 0.001; ^**^P < 0.01; and ^*^P < 0.05).

## Results

### Myricetin Alleviated Hepatic Steatosis, Hepatocytes Injury and Death, and Inflammation in a Diet-Induced Murine Model of NASH

To assess the effect of myricetin on diet-induced NASH, mice were fed separately a CDAHFD diet with vehicle or myricetin for 8 weeks. As shown in Table 1, there were remarkably differences in body weight (BW) and liver/body weight ratio between CDAHFD-fed mice and NCD-fed mice. However, compared with the vehicle-treated to NASH mice, myricetin-treated to NASH mice did not alter either BW or liver/body weight ratio. Also, there was no differences in liver weight between myricetin-treated NASH mice and vehicle-treated NASH mice (Table 1). Moreover, we found CDAHFD-fed induced an increase in the activity of ALT and AST, which are surrogate markers used to indicate hepatocytes injury and death. However, myricetin-treated to CDAHFD-fed mice showed a statistically relevant reduction the levels of serum ALT and AST when compared with vehicle-treated to those mice (Table 1).

**Table 1:** The impact of myricetin treatment on body weight, liver weight, and serum enzymes at 8 weeks in mice fed NCD or CDAHFD.

	**NCD** **(N = 8)**	**CDAHFD + Veh** **(N = 8)**	**CDAHFD + Myr** **(N = 8)**
Body weight (g)	26.95 ± 0.65	20.31 ± 0.42^a^	21.65 ± 0.56^a^
Liver weight (g)	1.35 ± 0.06	1.34 ± 0.04	1.36 ± 0.09
Liver/body weight ratio (%)	4.98 ± 0.12	6.31 ± 0.15^a^	6.74 ± 0.32^a^
Serum ALT (U/L)	10.1 ± 2.2	281.1 ± 7.7^a^	188.6 ± 14.9^a^^,^^c^
Serum AST (U/L)	18.5 ± 2.4	137.7 ± 10.8^a^	88.7 ± 9.4^a^^,^^b^

*Mice were fed a normal chow diet (NCD) or the choline-deficient, L-amino acid-defined, high-fat diet (CDAHFD) for 8 weeks and concomitantly treated with myricetin (100 mg/kg) or vehicle by daily gavage. Values are expressed as the mean ± SEM. ALT, alanine transaminase; AST, aspartate transaminase; Myr, myricetin; Veh, vehicle; N, sample size*.

a P < 0.001 vs. NCD;

b P < 0.01,

c *P < 0.001 vs. CDAHFD + Veh*.

**Figure 2:**
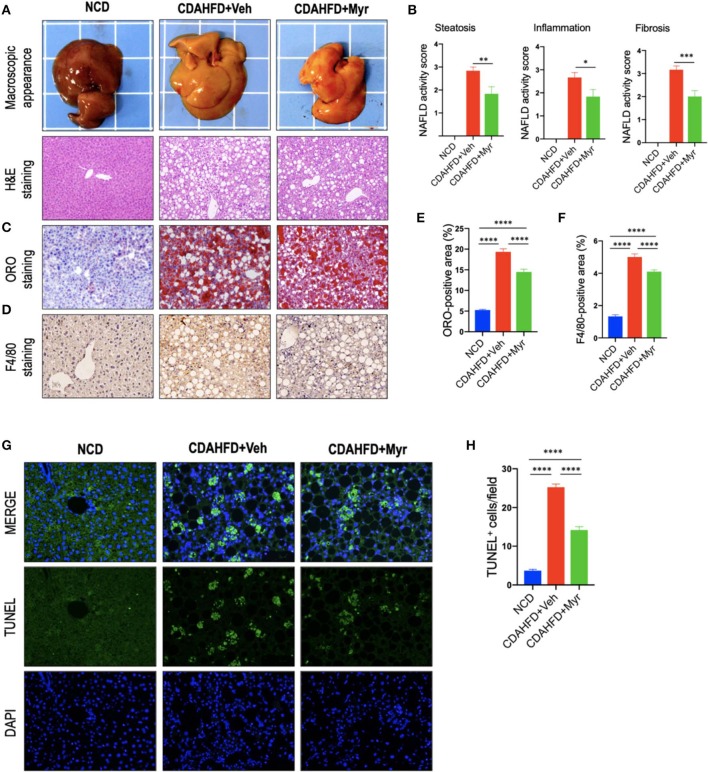
Myricetin (Myr) alleviated hepatic steatosis, hepatocytes injury, and death, and inflammation in a diet-induced murine model of NASH. **(A)** Representative macroscopic appearance of livers and H&E staining of the liver sections. Original magnification: ×100. **(B)** Histological analysis using NAFLD activity score (NAS). *N* = 6 for each group. **(C)** Oil Red O (ORO) staining of liver sections from mice. Original magnification: ×100. **(D)** Representative images of Immunohistochemical (IHC) staining of F4/80 in liver sections. Original magnification: ×200. **(E)** Quantification of ORO-positive staining. **(F)** Quantification of F4/80^+^ area staining in liver sections. Results mean of six fields and *n* = 5/group. **(G)** TUNEL staining for apoptotic cells of the liver sections from each group mice. Original magnification, ×200. **(H)** Quantification of the TUNEL^+^ cell number per high-power field (×200). *P < 0.05, **P < 0.01, ***P < 0.001, ****P < 0.0001.

Histological assessment exhibited that CDAHFD-fed mice highly induced lipid accumulation, hepatocyte death, and liver inflammation, with markedly enhanced macrophages infiltration that limited to the area surrounding the centrilobular veins of the liver (Figure 2). However, these morphological alterations were remarkably attenuated in NASH mice treated with myricetin. These observations were further confirmed by the NAFLD activity score from H&E-stained sections, which were lower in the myricetin-treated NASH mice than that in the vehicle-treated NASH mice (Figures 2A,B). As shown by ORO-staining, lipid accumulation in hepatocytes was also decreased in NASH mice with myricetin treatment when compared with the animals with vehicle treatment (Figures 2C,E). Moreover, hepatic macrophages were assessed by IHC staining for F4/80. As shown in Figure 2D, the basal amounts of hepatic macrophages were observed in NCD-fed mice, the number of hepatic macrophages and the foci containing macrophages were remarkably enhanced in mice fed with CDAHFD for 8 weeks. But treatment with myricetin obviously reduced the staining signaling of F4/80^+^ macrophages in CDAHFD-fed mice compared to vehicle-treated to those mice. These observation was further confirmed by quantification of the F4/80^+^ staining area, indicating that CDAHFD-fed for 8 weeks facilitated macrophages recruitment into the livers, and that the increased number of F4/80^+^ cells were remarkably decrease in myricetin-treated to NASH mice as compared with that in vehicle-treated to NASH mice (Figure 2F). In addition, we used TUNEL assay to evaluate the effect of myricetin on hepatocytes apoptosis in the liver from mice after feeding with CDAHFD for 8 weeks. As expected, the number of TUNEL^+^ cells were significantly increased in CDAHFD-fed mice compared that in NCD-fed mice; however, CDAHFD-fed mice receiving myricetin administration could lower the elevated number of TUNEL^+^ cells when compared to those animals receiving vehicle administration (14.16 ± 0.88/field vs. 25.24 ± 0.83/field; *P* < 0.0001; Figures 2G,H).

Taken together, our results demonstrated that myricetin alleviated hepatic steatosis, hepatocytes injury and death, and inflammation in a diet-induced murine model of NASH.

### Myricetin Inhibited Liver Fibrosis and HSC Activation in NASH Mice

**Figure 3:**
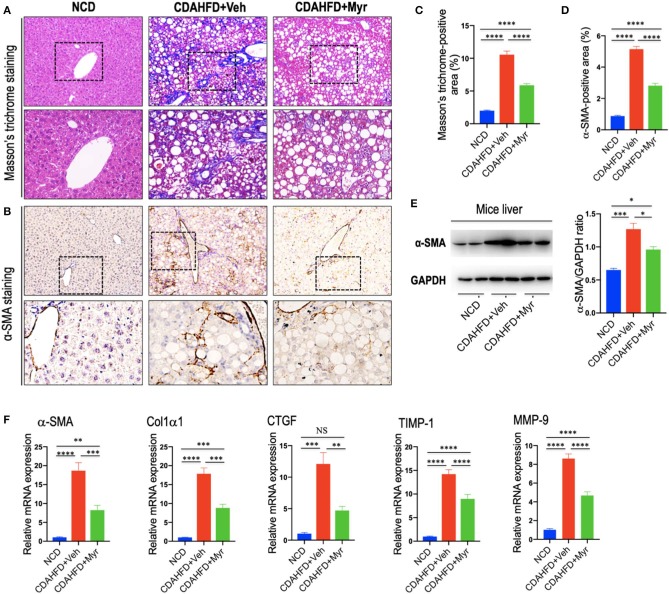
Myricetin inhibited liver fibrosis and HSC activation in NASH mice. **(A)** Masson's trichrome staining of liver sections in mice from fed an NCD or CDAHFD with myricetin (Myr) or Vehicle (Veh). Upper panel (original magnification, ×100) and lower panel (Original magnification, ×200). **(B)** Immunohistochemical (IHC) images of liver sections stained for α-smooth muscle actin (α-SMA). Bottom row contains images enlarged from the boxed area in the corresponding panel in the top row. Original magnification, ×100 (upper panel) and ×200 (lower panel). **(C)** Quantitative analysis of Masson's trichrome positive area. **(D)** Quantification of the α-SMA-positive area. **(E)** Western blotting analysis of α-SMA protein in livers; results were normalized relative to expression of GAPDH. **(F)** Transcript levels of fibrotic markers (α-SMA, Col1α1, CTGF, TIMP-1, and MMP-9) were measured by quantitative RT-PCR in whole liver samples (*n* = 5). Results were normalized to β-actin mRNA and expressed as folds change compared to NCD-fed mice. *P < 0.05, **P < 0.01, ***P < 0.001, ****P < 0.0001; “NS” indicates not significant.

As shown in Figure 3A, mice fed of CDAHFD for 8 weeks led to obviously collagen accumulation, with deposition of extracellular matrix and formation of thin portal-to-portal fibrous septa. In contrast, CDAHFD-fed mice with myricetin administration showed thinner septa and more preserved intact hepatocytes than those animals with vehicle administration (Figure 3A). These findings were further confirmed by the percentage of fibrotic areas from each section, which was remarkably decreased in CDAHFD-fed, myricetin-treated mice vs. vehicle-treated mice on the same diet (5.88% vs. 10.56%, *P* < 0.0001; Figure 3C). Similarly, the mean fibrosis score was significantly reduced in myricetin-treated NASH mice than that in vehicle-treated NASH mice (1.83 ± 0.31 vs. 2.83 ± 0.17, *P* = 0.009; Figure 2B). Consistent with these findings, we noted that the mRNA expression of profibrogenic markers, including collagen 1α1 (Col1α1), connective tissue growth factor (CTGF), matrix metalloproteinase-9 (MMP-9), and tissue inhibitor of metalloproteinase-1 (TIMP-1), were markedly increased in NASH livers than that in NCD-fed livers; however, myricetin administration remarkably abrogated the effect of CDAHFD and downregulated the expression of these profibrogenic markers (Figure 3F).

In order to investigate that effect of myricetin on HSC activation in the liver; we here examined the activated HSC marker α-SMA expression by IHC staining in liver sections. Our IHC showed that there was remarkably increased α-SMA immunostaining in the fibrotic septa in livers from CDAHFD-fed mice, while little staining of α-SMA in livers from NCD-fed mice; however, there was a relatively weak intensity in livers from myricetin-treated NASH mice when compared that from vehicle-treated NASH mice (Figure 3B). Consistent with this observation, semi-quantitative analysis demonstrated that the α-SMA-positive area was significantly lower in the liver from myricetin-treated NASH mice than those from vehicle-treated animals (2.82 ± 0.15% vs. 5.15 ± 0.18%, *P* < 0.0001; Figure 3D). These findings were further validated by western blotting and quantitative RT-PCR analysis that revealed lower expression α-SMA gene and protein in NASH mice with myricetin treatment compared with those mice with vehicle treatment (Figures 3E,F).

Collectively, these results indicated that myricetin strikingly attenuated NASH-related fibrosis and the activation of HSCs in a murine NASH model induced by CDAHFD.

### Myricetin Treatment Suppressed M1 Polarity Switch in the Liver Macrophages in NASH Mice

**Figure 4:**
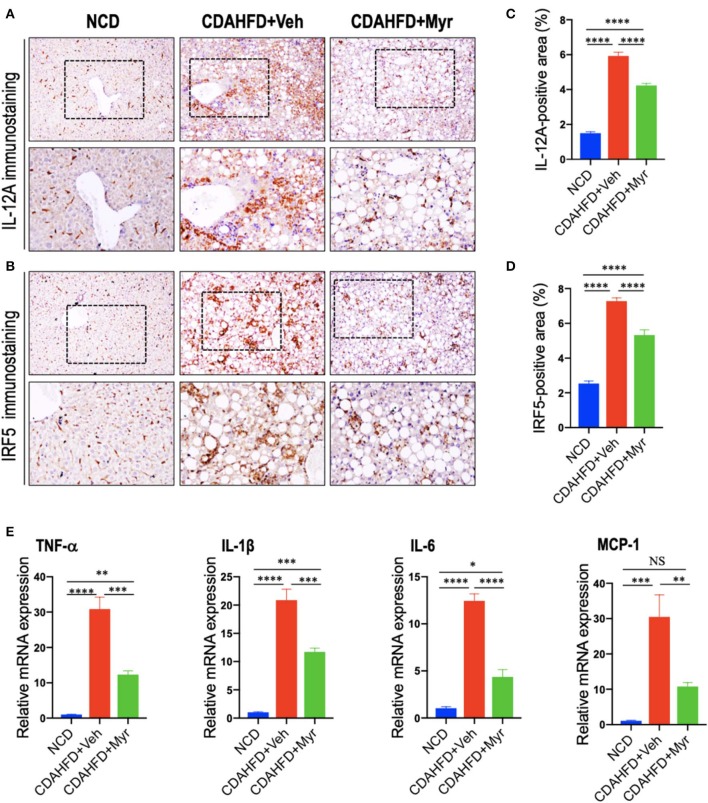
Myricetin treatment suppressed M1 polarity switch in the liver macrophages in NASH mice. **(A,B)** Representative immunostaining of IL-12A and interferon regulatory factor 5 (IRF5) in liver sections. Bottom row contains images enlarged from the boxed area in the corresponding panel in the top row. Original magnification: ×100 (upper panel) and ×200 (lower panel). **(C,D)** Quantification of the IL-12A^+^ and IRF5^+^ staining area in the liver from each group. **(E)** Hepatic mRNA expression of M1-polarized markers (TNF-α, IL-1β, IL-6, and MCP-1) was determined by quantitative RT-PCR, and results are shown as fold change compared with NCD-fed mice and β-actin served as loading control. *P < 0.05, **P < 0.01, ***P < 0.001, ****P < 0.0001; “NS” indicates not significant.

To determine whether myricetin limits hepatic inflammation and fibrosis by switching macrophages polarization and modulating their function, we assessed the effect of myricetin on M1 polarization and activation in the pathogenesis of NASH with fibrosis. Here, we selected IL-12A and interferon regulatory factor 5 (IRF5) as molecular markers of M1 polarization (5, 45). We found that CDAHFD-fed remarkably enhanced M1-polarized macrophages, as indicated by IHC staining of IL-12A and IRF5 (Figures 4A,B) and by analysis of the IL-12^+^ area and IRF-5^+^ areas in liver sections (Figures 4C,D); however, myricetin-treated NASH mice inhibited M1 macrophage phenotype as determined by M1-polarized markers. Of note, these positive macrophages were limited into hepatic sinusoids and fibrotic septa in NASH mice. These results were further confirmed by quantitative RT-PCR for M1-related markers and indicated that CDAHFD-fed mice were related to the increased proinflammatory cytokine markers in livers, including TNF-α, IL-1β, IL-6, and MCP-1 mRNA, as compared with the NCD-fed mice. However, compared with vehicle-treated to NASH diet mice, myricetin-treated to NASH diet mice reduced the levels of those M1 markers genetic expression (Figure 4E). Taken together, these results suggested that myricetin administration remarkably inhibited the M1-poliarized macrophages and reduced the expression of inflammatory properties in livers of NASH mice.

### Myricetin Treatment Enhanced M2 Polarity Switch in the Liver Macrophages in NASH Mice

**Figure 5:**
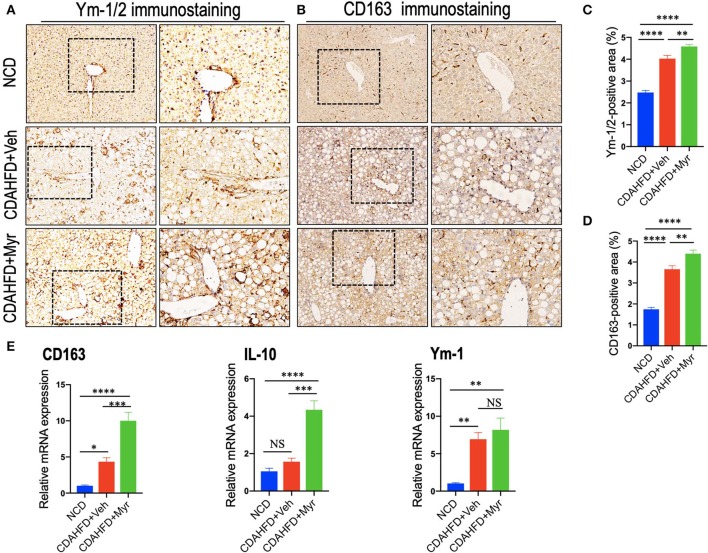
Myricetin (Myr) treatment enhanced M2 polarity switch in the liver macrophages in NASH mice. **(A,B)** Representative immunostaining of Ym-1/2 and CD163 in liver sections. Bottom row contains images enlarged from the boxed area in the corresponding panel in the top row. Original magnification: ×100 (left panel) and ×200 (right panel). **(C,D)** Quantification of the Ym-1/2^+^ and CD163^+^ staining area in livers from each group. **(E)** Hepatic mRNA expression of M2-polarized markers (CD163, IL-10, and Ym-1) was determined by quantitative RT-PCR, and the results are shown as folds change compared with NCD-fed mice and β-actin served as loading control (*n* = 5). *P < 0.05, **P < 0.01, ***P < 0.001, ****P < 0.0001; “NS” indicates not significant.

We also determined the effect of myricetin on M2-polarized macrophages in NASH mice with fibrosis. We applied chitinase-3-like 3 and 4 (also known as Ym-1 and Ym-2 in mice, respectively) and CD163 as molecular markers of M2-polarized macrophage (5, 45); and we noted that M2 macrophages in the liver were slightly increase in NASH mice induced by CDAHFD for 8 weeks as assessed by the Ym-1/Ym-2 and CD163 immunostaining (Figures 5A,B); however, NASH mice with myricetin treatment enhanced the density of Ym-1/Ym-2 and CD163 staining when compared with the NASH mice with vehicle treatment (Figures 5A,B). These results were further validated by percentages of Ym-1/2^+^ and CD163^+^ staining area (Figures 5C,D), suggesting myricetin-treated induced M2-polarized macrophages in livers from mice fed with CDAHFD. As a further corroboration, the M2 skewing was supported by the quantitative RT-PCR for selective M2-polarized markers such as CD163, IL-10, and Ym-1; and our results demonstrated that myricetin increased expression of CD163 and IL-10 mRNA, but there was no difference in Ym-1 gene expression between the myricetin-treated NASH mice and vehicle-treated NASH mice (Figure 5E).

Together, these results showed that myricetin treatment induced hepatic M2 macrophage polarization in CDAHFD-induced NASH and immunosuppressive genes (IL10 and CD163).

### Myricetin Treatment Suppressed M1-Polarized and Induced M2-Polarizd Macrophages *in vitro*

**Figure 6:**
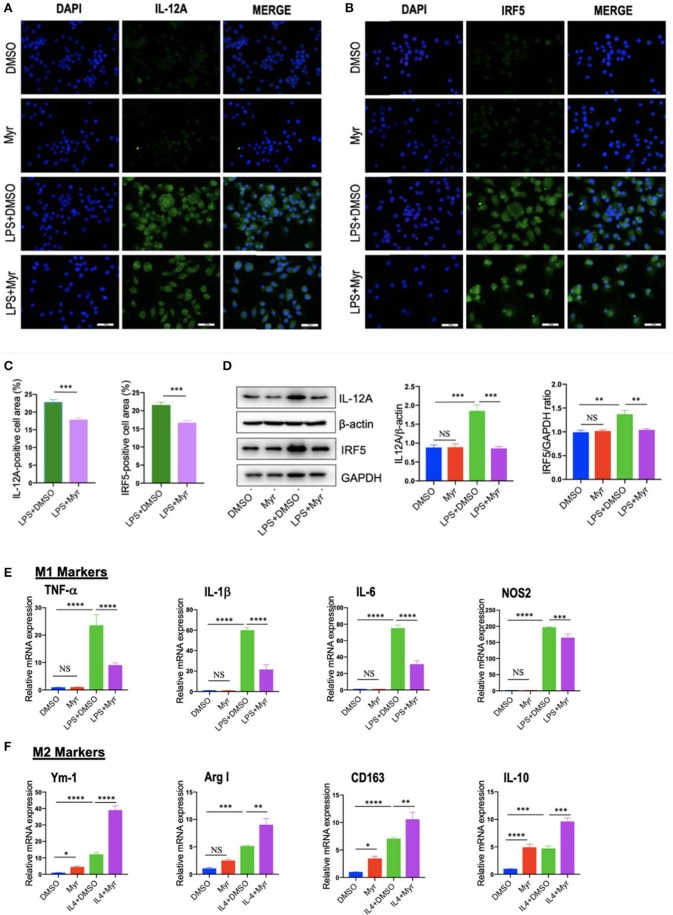
Myricetin (Myr) treatment suppressed M1 and induced M2 polarization of macrophages *in vitro*. **(A,B)** Representative fluorescence microscopic images of RAW264.7 cells with anti-IL12 and anti-IRF5 whole-mount staining. The nuclei were stained with 4′,6-diamidino-2-phenylindole (DAPI). Undifferentiated RAW macrophages conditioned medium (M0), M1-differentiated macrophages conditioned medium, Myr (50 μM) or DMSO treated M1-differentiated macrophages conditioned medium. Bars represent mean ± SEM of at least three independent experiments. Scale bar = 50 μm. **(C)** Percentage of the IL-12^+^ and IRF5^+^ staining area in 6 randomly selected fields. **(D)** Western blotting and quantification of M1-marker IL12 and IRF5 protein expression in macrophages RAW264.7 cells, with results normalized relative to the expression of β-actin or GAPDH, respectively (*n* = 3). **(E)** Quantification gene expression analysis of M1 markers TNF-α, IL-1β, IL-6, and NOS2. The mRNA levels were normalized to β-actin mRNA levels and presented as folds change vs. DMSO-treated control. **(F)** Quantification gene expression analysis of M2 markers Ym-1, Arg1, CD163, and IL-10. The mRNA level was normalized to β-actin mRNA level and presented as folds change vs. DMSO-treated control. *P < 0.05, **P < 0.01, ***P < 0.001, ****P < 0.0001; “NS” indicates not significant.

To investigate the effect of myricetin on macrophages polarization, we examined M1 and M2 markers by myricetin treatment in RAW264.7 cell induced by LPS or IL-4, respectively. As the RAW264.7 cell line can be reliably polarized to M1 phenotype *in vitro* by LPS stimulation (9, 11, 38), using the model of M1 macrophages, we found that administration with myricetin to the cells remarkably inhibited M1 polarity switch in macrophages as indicating in immunostaining with anti-IL12A and anti-IRF5 (Figures 6A–C). Next, we determined the expression of M1 macrophages markers such as IL12 and IRF5 by western blotting, our results revealed that myricetin administration markedly lowered the protein expression of those markers when compared with vehicle administration (Figure 6D). Moreover, our quantitative RT-PCR results also confirmed that myricetin treatment led to inhibiting M1 polarity switch in macrophages as depicted in mRNA expression of M1 markers (TNF-α, IL-1β, IL-6, and NOS2) (Figure 6E). Additionally, at this dosage, myricetin had not affect cell viability of macrophages *in vitro* (Figure S1).

On the other hand, to investigate M2 macrophages phenotypes switching by myricetin in RAW267.4 cells, the culture of RAW267.4 cells were administrated with vehicle, myricetin (50 μM), or IL-4 (20 ng/ml) for 12 h. We found that the mRNA expression of M2 marker (Ym-1, Arg1, CD163, and IL-10) was highly enhanced by administration with myricetin when compared with administration with vehicle (Figure 6F).

Collectively, these findings indicated that myricetin administration could suppress the M1-polarized macrophages and induce M2 polarity upon stimulation *in vitro*.

### Myricetin Treatment Inhibits the TREM-1-TLR2/4-MyD88 Signaling in the Liver of CDAHFD-Treated Mice and in LPS-Stimulated RAW264.7 Cells

**Figure 7:**
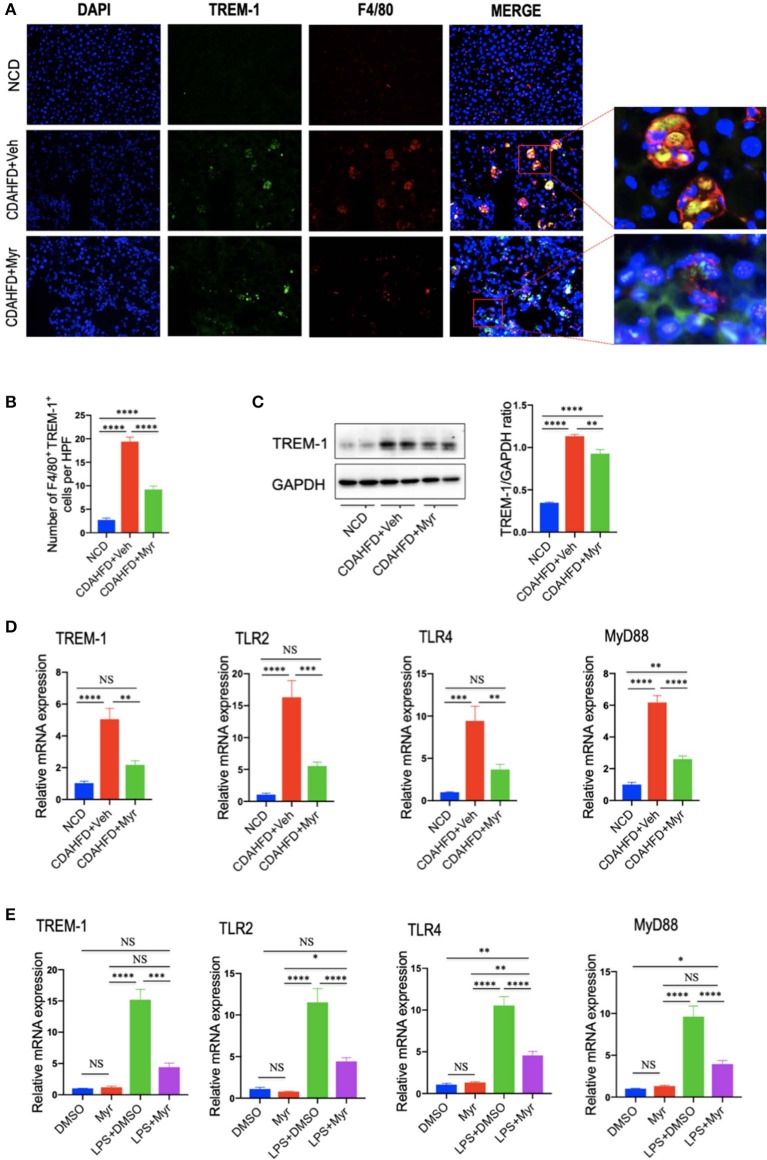
Myricetin treatment inhibits the TREM-1-TLR2/4-MyD88 signaling in the liver of CDAHFD-treated mice and in LPS-stimulated RAM267.4 cells. **(A)** Immunofluorescent double staining in liver sections from each group. Liver sections were double stained for TREM-1 (green) and F4/80 (Red) macrophages. Nuclei were stained with 4′,6-diamidino-2-phenylindole (DAPI) (blue). Original magnification: ×200; Scale bar = 50 μm. **(B)** Quantification of TREM-1 and F4/80 double-positive cells. **(C)** Western blotting and quantification of TREM-1 expression in lysed liver tissues, with results relative to the expression of GAPDH (*n* = 3). **(D)** Hepatic mRNA expression of TREM-1, TLR2, TLR4, and MyD88 was measured by quantitative RT-PCR. Results are shown as fold change compared with NCD-fed mice and β-actin served as loading control (*n* = 5). **(E)** The mRNA expression of TREM-1, TLR2, TLR4, and MyD88 in RAW264.7 cells were measured by quantitative RT-PCR. The mRNA levels were normalized to β-actin mRNA levels and presented as fold stimulation (mean ± SEM) vs. DMSO. *P < 0.05, **P < 0.01, ***P < 0.001, ****P < 0.0001; “NS” indicates not significant.

To further explore whether the decreased M1 macrophages by myricetin was due to inhibition TREM-1-mediated signaling, we firstly examined the TREM-1 expression and subcellular location in the liver. As shown in Figure 7A, immunofluorescence double staining of TREM-1 and F4/80 in liver sections from mice revealed that TREM-1^+^ and F4/80^+^ cells almost overlapped; and the staining signal of TREM-1 in NASH and fibrotic liver is stronger than NCD-treated liver. However, compared with vehicle-treated NASH mice, the double-staining signaling of the F4/80^+^ TREM-1^+^ cells were remarkably decreased. The number of double-positive TREM-1^+^ F4/80^+^ cells increased remarkably in NASH mice compared with normal control, but this increase was inhibited by myricetin treatment in NASH mice (Figure 7B). We further determined the gene and protein expression of TREM-1 in the liver from mice by quantitative RT-PCR and western blotting, respectively. Our results demonstrated that there was a remarkable increase in TREM-1 at both the mRNA and protein levels in livers from NASH as compared with those in livers from NCD-fed controls; however, myricetin-treated NASH mice decreased the TREM-1 expression both in gene and protein levels when compared with vehicle-treated NASH mice (Figures 7C,D). Additionally, we also determined the mRNA expression of TLR2, TLR4, and the adapter MyD88 in the liver; we also found that CDAHFD-fed induced those genes expression as compared with NCD-fed mice. However, the upregulated expression of those genes in NASH livers was significantly inhibited by myricetin treatment as compared with vehicle treatment (Figure 7D).

To better characterize whether myricetin inhibits macrophage polarization to M1 via regulating TREM-1-mediated signaling on macrophages, we investigated the expression of TREM-1, TLR2, TLR4, and MyD88 in RAW264.7 cells that incubated with myricetin prior to induction M1-polarized macrophages using LPS *in vitro*. We found that LPS remarkably enhanced the expression of TREM-1in RAW264.7 cells at gene level, whereas these elevated expression of TREM-1 was markedly abrogated by administration of 50 μM myricetin. Moreover, myricetin also significantly reduced the mRNA expression of TLR2, TLR4, and MyD88 in LPS-stimulated RAW264.7 cells relative to DMSO-treated cells (Figure 7E).

Taken together, these findings indicated that myricetin treatment inhibited TREM-1-TLR2/4-MyD88 signaling both *in vivo* and *in vitro* study, which might be, at least in part, involved in regulating liver macrophages polarization.

### Myricetin Inhibited the Activation of NF-κB Signaling and Reduced the Phosphorylation of STAT3 and JNK in LPS-Stimulated RAW264.7 Macrophages

**Figure 8:**
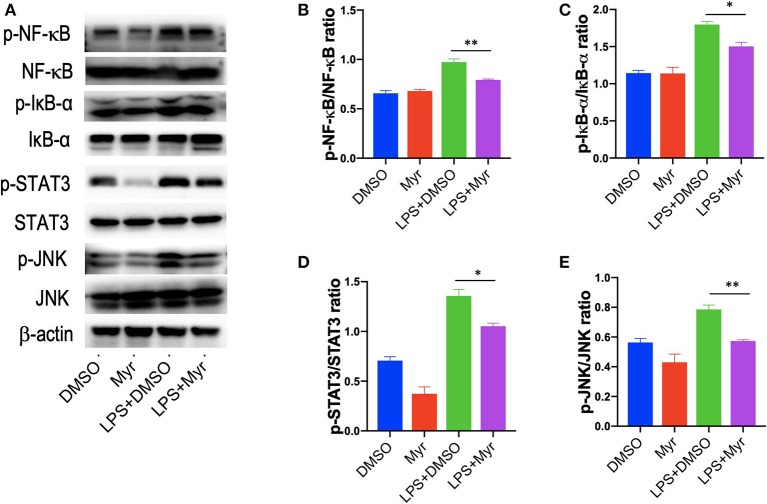
Myricetin inhibited the activation of NF-κB signaling and reduced the phosphorylation of STAT3 and JNK in LPS-stimulated RAW264.7 cells. RAW264.7 cells were pretreated with myricetin (50 μM) for 12 h and then incubated for 12 h with LPS (100 ng/mL). **(A)** Western blotting analysis of the p-NF-κB p65, NF-κB p65, p-Iκ-Bα, Iκ-Bα, p-STAT3, STAT3, p-JNK, and JNK levels in LPS-stimulated RAW264.7 macrophages; and β-actin protein levels served as control. **(B,C)** Quantification of p-NF-κB p65/ NF-κB p65 ratio and p-Iκ-Bα/ Iκ-Bα ratio. **(D,E)** Quantification of p-STAT3/STAT3 ratio and p-JNK/JNK ratio. *P < 0.05, **P < 0.01.

Since the transcription factor NF-κB plays a critical role in inflammatory response, and we explored the effects of myricetin on the activation of NF-κB pathways by assessing the phosphorylation of I-κBα and NF-κB in LPS-stimulated RAW 264.7 cells. Our results revealed that treatment of RAW264.7 cells with LPS induced an obviously increase of the phosphorylation of I-κBα and NF-κB, while application of myricetin inhibited the phosphorylated expression I-κBα and NF-κB (Figures 8A–C). Additionally, STAT3 and JNK signaling are progressively elevated in livers of NASH (7, 46), in this study, we found that p-STAT3 and p-JNK were also increased in LPS-stimulated RAW264.7 cells, however, myricetin pre-treated to LPS-stimulated RAW264.7 cells led to lower the increased expression of the p-STAT3 and p-JNK as compared with vehicle-treated to the cells (Figures 8A,C,D). Collectively, these finding indicated that myricetin inhibited LPS-induced TREM-1-TLR2/4-MyD88-mediated inflammatory response via inhibiting of NF-κB activation and reducing the phosphorylation of STAT3 and JNK expression.

## Discussion

Currently, there is no pharmacological agents that has been convincingly efficient against NASH and fibrogenesis (4, 47). In this study, we have provided both *in vivo* and *in vitro* evidence regarding the potent role of the myricetin in reducing the severity of steatosis, inflammation, hepatocyte cell injury and death, and fibrosis in CDAHFD-derived NASH model. Importantly, our results provide an evolving insight into the anti-inflammatory and antifibrotic effects of myricetin against NASH, which owning to its regulation the polarization of macrophages in the livers. Additionally, our data also have strongly reinforced the notion that liver macrophages polarization are associated with the development of NASH and fibrogenesis, and strategies inhibiting M1 polarity switch of macrophages may protect against exacerbated NASH and fibrosis (7, 9, 48).

Myricetin is one of the common plant-derived flavonoids, which exhibits a wide range of pharmacological effects including anti-inflammatory, antioxidant, anti-obesity, and antitumor activities (28–34, 37, 49). Here, using a murine model of diet-induced NASH, we demonstrated myricetin-treated to NASH mice significantly inhibited the pro-inflammatory cytokines (such as TNF-α, IL-1β, IL-6, and MCP-1) expression (Figure 4E). In line with decreased hepatic inflammation, plasma levels of ALT and AST were markedly decreased in myricetin-treated NASH mice (Table 1). Moreover, myricetin-treated to CDAHFD-fed mice markedly reduced hepatocyte apoptosis compared with vehicle-treated to CDAHFD-fed mice (Figures 2G,H). Meanwhile, our results also revealed that liver injury and hepatocytes death triggered the activation of Kupffer cell (Figure 2D), leading to inflammatory cytokine and chemokine production during NASH development. The perpetuation of inflammation and hepatocytes death further led to the pathogenesis of NASH and liver fibrosis (4, 5). However, myricetin treatment could inhibited this pathologic process. Indeed, our results confirmed that myricetin-treated to NASH mice effectively inhibited the activation of HSCs and hepatic fibrosis compared with vehicle-treated mice on the same diet (Figure 3). Supported our data, myricetin attenuated liver fibrosis-induced by CCl_4_ in mice (29, 32) and ameliorated high-fat diet-induced obesity and IR through enhancing antioxidant capacity (34). However, the exact molecular mechanisms by which myricetin exerts its beneficial effects on liver inflammation and fibrosis in NASH has been largely unknown.

Here, we used an established model of NASH to determine the mechanism by which myricetin improved liver disease. We highlighted the effects of myricetin on the activation and polarization of macrophages. Interestingly, we found that myricetin-treated to CDAHFD-fed mice suppressed M1-polarized macrophages in livers as shown by IHC staining of IRF5 and IL-12A and reduced mRNA expression of proinflammatory M1 marker (TNF-α, IL-1β, IL-6, and MCP-1) (Figure 4). Moreover, using murine macrophage RAW267.4 further confirmed that myricetin significantly inhibited LPS-induced M1-polarized macrophages *in vitro* (Figures 6A–E). It well documented that excessive or unresolved M1-polarized macrophages could incur chronic inflammation and tissue injury (39). Therefore, myricetin administration inhibited M1-polarized macrophages, which might be the mechanism of protection from NASH development and liver fibrogenesis exerted by myricetin.

On the other hand, we also assessed the effect of myricetin on M2 polarity switch of macrophages in NASH and fibrosis in mice. Our IHC results showed that M2 macrophage markers, Ym-1/2^+^ and CD163^+^, were both remarkably increased at 8-week CDAHFD-fed when compared with NCD-fed controls; however, myricetin treatment further induced the numbers of Ym-1/2^+^ cells and CD163^+^ cells. We also found hepatic mRNA expression of M2 markers (IL-10, CD163, and Ym-1) in NASH mice were induced as compared to that in NCD-fed control mice (Figure 5); however, myricetin-treated to NASH mice further increased hepatic mRNA expression of CD163 and IL-10 markers when compared with vehicle-treated to those animals, but there was no difference in Ym-1 gene expression (Figure 5). *In vitro* experiment, using IL-4 induced M2 polarization, we found myricetin induced M2-polarized macrophage genes (Ym-1, Arg1, CD163, and IL-10) similarly IL-4-induced macrophages (Figure 6F). Noticeably, several previous studies have also demonstrated that M2 macrophages activation (Ym-1^+^, CD206^+^, or CD163^+^) was induced and had the potential effect on inflammatory response and fibrogenesis both in chronic liver diseases and in animal models (47, 50–52). However, it's worth to note that M2 macrophages significantly decreased at the later stage of NASH and fibrosis (51). Although M2 polarity of macrophages is often thought to having anti-inflammatory or reparative properties, excessive or unrestricted M2-polarized macrophage can also result in immune dysregulation and liver fibrosis (38, 39, 52). Therefore, our results provided a new insight for understanding the anti-inflammatory and antifibrotic effect of myricetin in NASH, which owning to its attenuation of liver macrophages infiltration and suppression of the M1 polarity of liver macrophages.

Importantly, our results further revealed that the molecular mechanism of modulation of macrophages polarization in livers by myricetin was surmised to be direct inhibition of the TREM-1-TLR2/4-MyD88 signaling pathways (Figure 7). Our immunofluorescent double staining result indicated that the gene and protein expression of TREM-1 on macrophages was upregulated in the liver of NASH mice together with quantitative RT-PCR and Western blotting. However, myricetin treatment abrogated the increase levels of TREM-1 gene and protein expression (Figures 7A–D). Activation of TREM-1 has been shown to trigger and aggravate inflammation, especially through synergism with TLRs signaling (13–16); therefore, we further assessed the effects of myricetin on the mRNA expression of TLR2, TLR4, and its adapter protein MyD88. As expected, our result confirmed that myricetin treatment significantly inhibited TLR2/4-MyD88 signaling expression in livers from NASH mice. Thus, TREM-1-mediated inflammatory response at least in part was associated with hepatic macrophages polarization in NASH. Indeed, previous data have also revealed that TREM-1 pathway plays an important role in macrophage-mediated inflammatory response (18–21). Interestingly, a recent study suggested that TREM-1 receptor also mediated in reversing M2 polarization induced by hypoxia (18). Although the specific ligands of TREM-1 have not been identified yet, it has been revealed that TREM-1 activation amplifies the TLR2/4-mediated proinflammatory signals, allowing the secretion of proinflammatory chemokines and cytokine (14, 17). Moreover, recent studies have demonstrated that TREM-1 mediated signaling modulation of M1 macrophage activation promoted the inflammatory response in alcoholic liver disease and obesity-induced fatty liver disease (18, 20). To further delineate how myricetin switches macrophage polarity in NASH development, we examined TREM-1-mdiated inflammatory responses *in vitro* of LPS-induced macrophage activation, consistent with M1 macrophage polarization, the expression of TREM-1 was upregulated on LPS-induced macrophages. However, treatment of myricetin attenuated M1 polarity switch of macrophages and that was associated with the reduce of TREM-1 signaling expression on macrophages (Figure 7). Given that TLR2/4 signaling pathway also contribute to proinflammatory macrophages activation (53, 54), we deduced that this inhibitory effect of myricetin may be also involved in blocking M1 macrophages polarization. Taken together, our data highlighted an important role for the TREM-1-TLR2/4 signaling pathways in regulating M1 macrophage polarization in CDAHFD-fed induced NASH and liver fibrosis, indicating that inhibition of TREM-1 signaling might be an effective therapeutic target for NASH and liver fibrosis.

Of note, our results further revealed that myricetin led to inhibiting LPS-induced TREM-1-mediated inflammatory response via downregulation of NF-κB activation and reducing the phosphorylation of STAT3 and JNK expression as shown by our western blotting (Figure 8). These findings were consistent with previous studies exhibiting that myricetin attenuated inflammatory response in LPS-induced macrophages *in vitro* and in streptozotocin-induced diabetic nephropathy by inhibiting NF-κB signaling pathways (28, 37).

In conclusion, administration of myricetin attenuated hepatocyte injury and death, inflammation, and fibrogenesis in the CDAHFD-diet-induced NASH model through regulating polarization of macrophages in livers via TREM-1-TLR2/4-MyD88 signaling pathways. These results suggested that myricetin could be considered a potential therapeutic agent for NASH and liver fibrosis.

## Data Availability Statement

The raw data supporting the conclusions of this article will be made available by the authors, without undue reservation, to any qualified researcher.

## Ethics Statement

The animal study was reviewed and approved by The Animal Ethics Committee of Zhongshan hospital.

## Author Contributions

QY, SL, and CT conceived the study and wrote the manuscript. XL, QY, FW, and CT contributed to the work designing, performing, analyzing, and interpreting data from all the experiments. QY, SL, FW, and CT participated in the design, acquisition, analysis, and interpretation of data. CT, XL, and SL carried out the animal model and all the *in vivo* animal experiments. CT, QY, and SL interpreted the data and finalized the article. All authors have critically revised and approved the final manuscript and agreed to be accountable for all aspects of the work.

### Conflict of Interest

The authors declare that the research was conducted in the absence of any commercial or financial relationships that could be construed as a potential conflict of interest.
