# Supplementary material for: Myricetin Modulates Macrophage Polarization and Mitigates Liver Inflammation and Fibrosis in a Murine Model of Nonalcoholic Steatohepatitis
Source: Front Med (Lausanne). 2020 Mar 4;7:71. doi: 10.3389/fmed.2020.00071 (PMC7065264; doi:10.3389/fmed.2020.00071)
Supplement: Supplementary file 1 [file Data_Sheet_1.docx]

Myricetin modulates macrophage polarization and mitigates liver inflammation and fibrosis in a murine model of nonalcoholic steatohepatitis

Qunyan Yao^1, 2, †^, Shuyu Li^1, 2, †^, Xi Li^3^, Fu Wang^4^ and Chuantao Tu^5,^ *

**Supplementary**

**Figure S1** RAW 264.7 cells were treated with LPS and various concentrations of MH (25, 50, 100 μM) for 0, 12, 24, 48 h, and cell viability was assessed by MTT assay. Cell viability was defined relative to no treatment control (NC), and the experiments were conducted three times independently.

**Table S1** List of primers used in this study.

| Target gene | Forward primers (5'-3') | Reverse primers (5'-3') |
| --- | --- | --- |
| CTGF | GCGCCTGTTCTAAGACCTGT | TTCATGATCTCGCCATCGGG |
| Col1α1 | GCTCCTCTTAGGGGCCACT | CCACGTCTCACCATTGGGG |
| TREM-1 | GACTGCTGTGCGTGTTCTTTG | GCCAAGCCTTCTGGCTGTT |
| TIMP-1 | CCAGAACCGCAGTGAAGAGT | TCTGGTAGTCCTCAGAGCCC |
| MMP-9 | AAAGGCAGCGTTAGCCAGAA | ACAACTCGTCGTCGTCGAAA |
| α-SMA | GTCCCAGACATCAGGGAGTAA | TCGGATACTTCAGCGTCAGGA |
| Arg I | CTCCAAGCCAAAGTCCTTAGAG | AGGAGCTGTCATTAGGGACATC |
| Ym-1 | CAGGTCTGGCAATTCTTCTGAA | GTCTTGCTCATGTGTGTAAGTGA |
| MyD88 | AGGACAAACGCCGGAACTTT | GCCGATAGTCTGTCTGTTCTAGT |
| TLR2 | TCTGATGGTGAAGGTTGGA | TGCTGAAGAGGACTGTTATG |
| TLR4 | CAGAACAATAGAAGAGGAAGAC | GGCACTAACCACATAGAGAA |
| TNF-α | CCCTCACACTCAGATCATCTTCT | GCTACGACGTGGGCTACAG |
| IL-1β | GCAACTGTTCCTGAACTCAACT | ATCTTTTGGGGTCCGTCAACT |
| IL-10 | GCTCTTACTGACTGGCATGAG | CGCAGCTCTAGGAGCATGTG |
| IL-6 | GGAGTCACAGAAGGAGTGGC | CGCACTAGGTTTGCCGAGTA |
| MCP-1 | AGCCAACTCTCACTGAAGCC | GGACCCATTCCTTCTTGGGG |
| CD163 | ATGGGTGGACACAGAATGGTT | CAGGAGCGTTAGTGACAGCAG |
| NOS2 | GAGCAACTACTGCTGGTGGT | CGATGTCATGAGCAAAGGCG |
| β-Actin | GTGACGTTGACATCCGTAAAGA | GTGACGTTGACATCCGTAAAGA |
